# Association between congenital heart disease and autism spectrum disorders: A protocol for a systematic review and meta-analysis

**DOI:** 10.1097/MD.0000000000033247

**Published:** 2023-03-17

**Authors:** Dan Ma, Jing-Lan Huang, Tao Xiong

**Affiliations:** a Department of Rehabilitation Medicine, West China Second University Hospital, Sichuan University, Chengdu, China; b Department of Pediatrics, West China Second University Hospital, Sichuan University, Chengdu, China; c Key Laboratory of Birth Defects and Related Diseases of Women and Children (Sichuan University) Ministry of Education, Chengdu, China.

**Keywords:** autism spectrum disorder, congenital heart disease, meta-analysis, protocol, systematic review

## Abstract

Congenital heart disease (CHD), the most common heart defect in children, refers to congenital disease with abnormal development of the heart or large blood vessels during the fetal period. The researchers suggest that children with CHD show more obvious neurodevelopmental disorders than children with normal development, and children with CHD may have a higher risk of social interaction and communication disorders. This is similar to the characteristics of children with autism spectrum disorder (ASD). However, the association between type of CHD and ASD is not well understood. This systematic review and meta-analysis will reveal the relationship between type of CHD and ASD.

**Methods:**

We will search the Cochrane Library, Embase, PubMed, China National Knowledge Infrastructure, Wanfang, Chinese Scientific Journals Full text, and China Biology Medicine disc databases using relevant subject terms and free words. We will use a fixed effects model or random effects model for meta-analysis. The risk of bias will be assessed by the Newcastle–Ottawa Scale and the agency for health care research and quality. Heterogeneity will be tested by *Q* statistics and *I*² values. Publication bias will be detected by funnel plots and Egger test. Subgroup analyses and sensitivity analyses will also be used to explore and interpret the heterogeneity.

**Results:**

The study will afford additional insight into the investigation the association between type of CHD and ASD.

**Conclusions:**

The results will provide evidence for the early identification and early intervention of ASD in children with CHD, which may contribute to improving the neurodevelopmental outcome of children with CHD.

## 1. Introduction

Congenital heart disease (CHD) refers to a congenital disease with structural or functional abnormal development of the heart or large blood vessels during the fetal period.^[1]^ CHD is one of the most common birth defects and a leading cause of death in infancy.^[2]^ In recent years, the prevalence of CHD has increased significantly worldwide.^[3]^ The reported prevalence of CHD ranges from 4 to 50 per 1000 live births.^[4]^ Approximately 1 in 4 infants with heart defects has critical CHD.^[5]^ Infants with critical CHD need surgery or other procedures in the first year of life.^[6]^ The increasing number of patients with CHD not only increases the demand for medical services but also places a heavy burden on families.

Currently, the causes of CHD are not completely known. CHD may be associated with many factors, including genetic factors, environmental factors, and uterine factors during pregnancy.^[7]^Genetic factors may play an important role in the pathogenesis of CHD.^[8]^ Related studies have shown that mutations or deletions of some genes on chromosomes not only lead to CHD but are also are related to neurodevelopmental disorders.^[9,10]^For example, the microdeletion of 22q11.2 can lead to CHD and neurodevelopmental disorders.^[11]^Some epidemiological studies have reported that children with CHD show more obvious neurodevelopmental disorders than children with normal cardiovascular development, and children with CHD may have a higher risk of social interaction and communication disorders.^[12,13]^ This is similar to the characteristics of children with autism spectrum disorder (ASD).

ASD is classified as a neurodevelopmental disorder as defined in DSM-5 (Diagnostic and Statistical Manual of Mental Disorders, 5th Edition) by the American Psychiatric Association.^[14]^ ASD is characterized by impairments in 3 behavioral domains, including social interaction, communication and stereotyped repetitive behaviors.^[15]^ In the 2010 Global Burden of Disease study, an estimated 52 million people had ASD globally, equating to a prevalence of 1 in 132 individuals.^[16]^ Epidemiological studies have demonstrated that ASD is more common in males than in females, with reported ratios ranging from 2:1 to 5:1.^[17,18]^Although the diagnosis of ASD is often established in childhood, ASD-related neurological impairments are generally lifelong. Thus, ASD not only has been regarded as a major public health challenge worldwide but also represents a substantial economic burden, which results in higher health care and school costs and loss of income for caregivers.

The etiology of ASD is not clear. Studies have shown that it may be related to genetic factors, abnormal brain structure and abnormal neuro bio chemistry. ^[19–21]^ Additionally, ASD is often accompanied by other disorders, including CHD.^[22–24]^ Whether CHD is a concurrent risk factor for ASD remains unclear. Although a study has shown that congenital heart disease may increase the risk of autism, but this study did not include a subgroup analysis of congenital heart disease types.^[25]^ Are there differences in the risk of autism spectrum disorders by type of CHD (atrial septal defect, ventricular septal defect, patent ductus arteriosus, tetralogy of Fallot, pulmonary valve stenosis, and other critical congenital heart defects)? Therefore, this systematic review and meta-analysis will be performed to summarize the available evidence regarding the relationship between type of CHD and ASD. Identifying relationships between type of CHD and ASD could help us to understand the pathogenesis of ASD. It also facilitates the early recognition and intervention of ASD in high-risk populations.

## 2. Methods

This study protocol followed the recommendations by the preferred reporting items for systematic reviews and meta-analyses protocols ^[26]^ and Meta-analysis of Observational Studies in Epidemiology guidelines to complete systematic reviews and meta-analysis.^[27]^This study protocol has been registered with the International Prospective Register of Systematic Reviews (CRD42020214364).

### 2.1. Study characteristics

#### 2.1.1. Population.

People at any age with CHD, with no ethnicity or status restrictions.

#### 2.1.2. Intervention/exposures.

Any types of CHD, such as atrial septal defect, ventricular septal defect, patent ductus arteriosus, tetralogy of Fallot, pulmonary valve stenosis, transposition of great arteries, coarctation of the aorta and hypoplastic leftheart syndrome.^[22]^

#### 2.1.3. Comparison.

People at any age without CHD.

#### 2.1.4. Outcomes.

Primary outcome: Diagnosed with ASD

1. A categorical diagnosis of ASD according to the DSM (III, III-R, IV, IV-TR or V) or International Statistical Classification of Diseases and Related Health Problems-9/10/11th (ICD-9,10,11), including previous categories: autistic disorder, Asperger syndrome or pervasive developmental disorder not otherwise specified.
2. A diagnosis was based on the Autism Diagnostic Observation Schedule and Autism Diagnostic Interview Scale-Revision. These diagnostic tools have good reliability and validity and are widely used worldwide.

Secondary outcome: Severity of ASD

Severity of ASD according to the DSM-V, which is divided into 3 levels:

1. Requiring very substantial support (Level 3),
2. Requiring substantial support (Level 2),
3. Requiring support (Level 1).

### 2.2. Eligibility criteria

#### 2.2.1. Inclusion criteria.

1. We will include cohort, case–control or cross-sectional studies in which a diagnosis of CHD and ASD are the outcome of interest.
2. We will include studies estimating the relationship between type of CHD and ASD and reporting the risk estimated odds ratios (ORs) and their corresponding 95% confidence intervals (CIs) (or data to calculate them).
3. We will include studies in English and Chinese.
4. No publication year restriction.
5. Studies where the participants are human.

#### 2.2.2. Exclusion criteria.

1. Review papers, conference abstracts, presentation and non-peer-reviewed government/local reports.
2. Studies with overlapping data.
3. Studies missing raw data.

### 2.3. Data sources and search strategy

We will search the Cochrane Library, Embase, PubMed, China National Knowledge Infrastructure, Wanfang, Chinese Scientific Journals Fulltext, and China Biology Medicine disc databases using relevant subject terms and key words. The detailed search strategy is listed in online (see Table S1, Supplemental Digital Content, http://links.lww.com/MD/I654, Search strategy used in PubMed database.)

We will manually search the references lists of the included studies, review papers, and conference summaries to identify additional studies. Furthermore, the search will be performed again prior to final analysis to identify additional eligible studies. A 3-step search strategy will be performed in this review.

1. For CHD, the following combination of search terms will be used:

“congenital heart disease” OR “congenital heart defect” OR “congenital heart malformation” OR “congenital heart anomalies” OR “CHD” OR” congenital cardiac malformation “ OR “congenital cardiac anomalies” OR “congenital cardiovascular disease” OR “congenital cardiac disease” OR “congenital cardiac defect” OR “cardiovascular malformation” OR “cardiovascular defect” OR “cardiovascular anomalies” OR “atrial septal defect” OR “ventricular septal defect” OR “patent ductus arteriosus “OR “ tetralogy of Fallot “ OR “ pulmonary valve stenosis “OR “transposition of great arteries” OR “ coarctation of the aorta “ OR “ hypoplastic left heart syndrome.”

1. For ASD, the following combination of search terms will be used: “autism spectrum disorder” OR “Autism” OR “ASD” OR “Kanner syndrome” OR “Kanner syndrome” OR “Autistic traits” OR “Infantile Autism” OR “Autistic disorder” OR “Asperger disorder” OR “Asperger syndrome” OR “Pervasive developmental disorder” OR “PDD.”
2. We will combine steps 1 and 2 with “AND.”

### 2.4. Data management

#### 2.4.1. Study selection and data extraction.

Study selection will be performed by 2 authors independently. The search results from these electronic databases and additional trials from other resources will be sent to Endnote X9. Two researchers will filter the title and/or search the study summary using a selection strategy to determine possible inclusion. If there is disagreement on inclusion, a third researcher will be consulted. After inclusion, the following extraction will be performed: generation information of the study (title, author, year, country, etc), population (number, baseline characteristics, diagnosis criteria, etc), exposure (CHD), outcomes (ASD), study characteristics (design, etc). All data included in the study will be extracted using a prespecified data form listed in online (see Table S2, Supplemental Digital Content, http://links.lww.com/MD/I655, Data Extraction Form)

#### 2.4.2. Data synthesis.

The Cochrane Collaboration Rev Man 5.3 (https://training.cochrane.org/online-learning/core-software/revman) and Stata V.14.0 (Statacorp) will be used for statistical analysis. The relationship between type of CHD and risk of ASD was expressed using OR and 95% CI. Both the crude and adjusted OR and the 95% CI will be calculated for the dichotomous variables. A *P* < .05 will be considered to be statistically significant. Heterogeneity will be assessed by the χ2 test and the *I*^2^ test. We will use a fixed effects model or random effects model for meta-analysis. The fixed effects model will be used when the *I*² value < 50% and *P* value > .1; otherwise, the random effects model will be selected.

#### 2.4.3. Subgroups/subsets analysis.

If sufficient data are available, the subgroup analysis will be based on; Study design; Types of CHD; Number of surgeries; Severity of CHD (cardiac murmur, cyanosis, arrhythmias, oxygen saturation); Severity of ASD (lever 1, 2, 3); Prognosis of ASD, and; Other characteristics of participants and exposure, which influence CHD and ASD.

#### 2.4.4. Sensitivity analysis.

Sensitivity analysis will be carried out based on the sample size, the missing data results and the methodological quality of the included study. If necessary, we will exclude studies with a high/medium risk of bias or other special characteristics to evaluate the stability of the results.

#### 2.4.5. Risk of bias (quality) assessment.

The Newcastle–Ottawa Scale will be used to assess the quality of cohort and case–control studies.^[28]^ The Newcastle–Ottawa Scale consists of 8 question items under 3 broad dimensions, including selection of study groups, comparability, and exposure or outcome. It adopts the semi quantization principle of the star system to evaluate the literature quality, and the full score is 9 stars. A high score represents a low risk of bias. Additionally, the Agency for Health care Research and Quality recommended items scale will be used to assess the quality of cross-sectional studies.^[29]^

### 2.5. Confidence in cumulative evidence

The quality of evidence will be assessed based on the Grading of Recommendations, Assessment, Development, and Evaluation system and will be classified into 4 levels: high, moderate, low, or very low.^[30]^ Evidence is rated based on Grading of Recommendations, Assessment, Development, and Evaluation 5 criteria for downgrading: risk of bias, inconsistency, indirectness, inaccuracy and publication bias. Two researchers will independently perform the quality assessment of each study, and any disagreements will be resolved through discussion or with a third author when necessary. If more than 10 studies are included, funnel plots will be used to check for reporting bias. Egger linear regression will be used to detect funnel plot asymmetry.

### 2.6. Presenting and reporting the results

The process of study selection will be summarized in a PRISMA flow diagram (see Figure S1, Supplemental Digital Content, http://links.lww.com/MD/I656, PRISMA flow diagram.). Additionally, the reason for studies being excluded from the full text will be noted. We will list in detail the characteristics of each included study, including the population, age range of participants, sample size, year range of studies, study designs, countries, exposure, outcome, measure of effect, unadjusted or adjusted effect (95% CI), and adjustment for covariates. The forest map will be used in the meta-analysis to represent the pooled estimates. For the eligible studies without enough raw data, we will contact corresponding authors. The information will be listed individually in a separate table.

### 2.7. Ethics and dissemination

The data for this study are from previously reported studies; therefore, no ethical approval was needed. The findings of this study will be presented at scientific conferences and published in a peer-reviewed journal.

## 3. Results

The results of this systematic review will be published as a peer-reviewed article.

## 4. Conclusions

This systematic review and meta-analysis will summarize the available literature exploring the association between types of CHD and the risk of ASD based on observational studies. This study will determine whether differences in the risk of ASD by type of CHD and whether CHD affects the severity of ASD. In addition, this study will help pediatricians provide useful information for neurodevelopmental monitoring in children with CHD. The results will provide evidence for the early identification and early intervention of ASD in children with CHD, which may contribute to improving the neurodevelopmental outcome of children with CHD.

However, this study has some limitations. Language bias may exist because only studies published in English and Chinese will be considered.

## Acknowledgments

J-LH and TX contributed equally to the correspondence work.

## Author contributions

**Conceptualization:** Dan Ma, Jing-Lan Huang.

**Data curation:** Jing-Lan Huang.

**Formal analysis:** Dan Ma.

**Investigation:** Dan Ma.

**Methodology:** Jing-Lan Huang.

**Project administration:** Tao Xiong.

**Software:** Jing-Lan Huang.

**Supervision:** Tao Xiong.

**Validation:** Dan Ma.

**Visualization:** Jing-Lan Huang.

**Writing – original draft:** Dan Ma.

**Writing – review & editing:** Tao Xiong.

## Supplementary Material
