# Supplementary material for: Association between congenital heart disease and autism spectrum disorders: A protocol for a systematic review and meta-analysis
Source: Medicine (Baltimore). 2023 Mar 17;102(11):e33247. doi: 10.1097/MD.0000000000033247 (PMC10019193; doi:10.1097/MD.0000000000033247)
Supplement: Supplementary file 1 [file medi-102-e33247-s001.pdf]

Table S1 Search strategy used in PubMed database

#1	Search terms	No of records returned
1	congenital heart disease	
2	congenital heart defect	
3	congenital heart malformation	
4	congenital heart anomalies	
5	CHD	
6	congenital cardiac malformation	
7	congenital cardiac anomalies	
8	congenital cardiovascular disease	
9	congenital cardiac disease	
10	congenital cardiac defect	
11	cardiovascular malformation	
12	cardiovascular defect	
13	cardiovascular anomalies	
14	atrial septal defect	
15	ventricular septal defect	
16	patent ductus arteriosus	
17	tetralogy of Fallot	
18	pulmonary valve stenosis	
19	transposition of great arteries	
20	coarctation of the aorta	
21	hypoplastic leftheart syndrome	
22	(#1 OR #2 OR #3 OR #4 OR #5 OR #6 OR #7 OR #8 OR #9 OR #10 OR #11 OR #12 OR #13 OR #14OR #15OR #16OR #17OR #18OR #19OR #20OR#21)	
23	Autism Spectrum Disorder	
24	Autism	
25	ASD	
26	Kanner's Syndrome	

27	Kanner Syndrome	
28	Autistic traits	
29	Infantile Autism	
30	Autistic Disorder	
31	Asperger Disorder	
32	Asperger syndrome	
33	Pervasive Developmental Disorder	
34	PDD	
35	(#23OR #24 OR #25 OR #26 OR #27 OR #28 OR #29 OR #30OR #31 OR #32 OR #33OR #34)	
36	(#22 AND #35)	
37	limit #36 to English and Chinese language	
38	limit #37 to humans	
