# Supplementary material for: Association between congenital heart disease and autism spectrum disorders: A protocol for a systematic review and meta-analysis
Source: Medicine (Baltimore). 2023 Mar 17;102(11):e33247. doi: 10.1097/MD.0000000000033247 (PMC10019193; doi:10.1097/MD.0000000000033247)
Supplement: Supplementary file 2 [file medi-102-e33247-s002.pdf]

Table S2. Data Extraction Form for Review and meta-analysis

Study Details	
**General information**	
• First author	
• Year of publication	
• Study location	
• Study duration	
**Study eligibility**	
• Study design (case-control, cohort)	
• Participants	
exposed group	
unexposed group	
• Inclusion criteria	
• Exclusion criteria	
• Ascertainment of exposure	
• Outcome diagnostic criteria	
• Confounding variables	
• Matching factors	
Include or exclude	Include ☐ exclude ☐
Reason for exclusion	
**Characteristics of included studies**	
• Sample size	
exposed group (including subgroups)	
unexposed group	
• Data source	
• Race	
• Income level of country	
• CHD`s adjusted factors	
types of CHD	
severity of CHD	
number of surgeries	

cardiac murmur	
cyanosis, arrhythmias	
oxygen saturation	
Others	
• ASD`s adjusted factors	
severity of ASD	
prognosis of ASD	
Others	
**Main outcome**	
Effect estimates (RR or OR) with 95% CI	
Mean difference	
Other findings	

Other information

	Description as stated in report/paper
Key conclusions	
Study funding sources	
Conflicts of interest	
References to other relevant studies	
Correspondence required for further study information	
