# Supplementary figures and images for: Association between congenital heart disease and autism spectrum disorders: A protocol for a systematic review and meta-analysis
Source: Medicine (Baltimore). 2023 Mar 17;102(11):e33247. doi: 10.1097/MD.0000000000033247 (PMC10019193; doi:10.1097/MD.0000000000033247)

figure S1

Flow diagram of study selection process

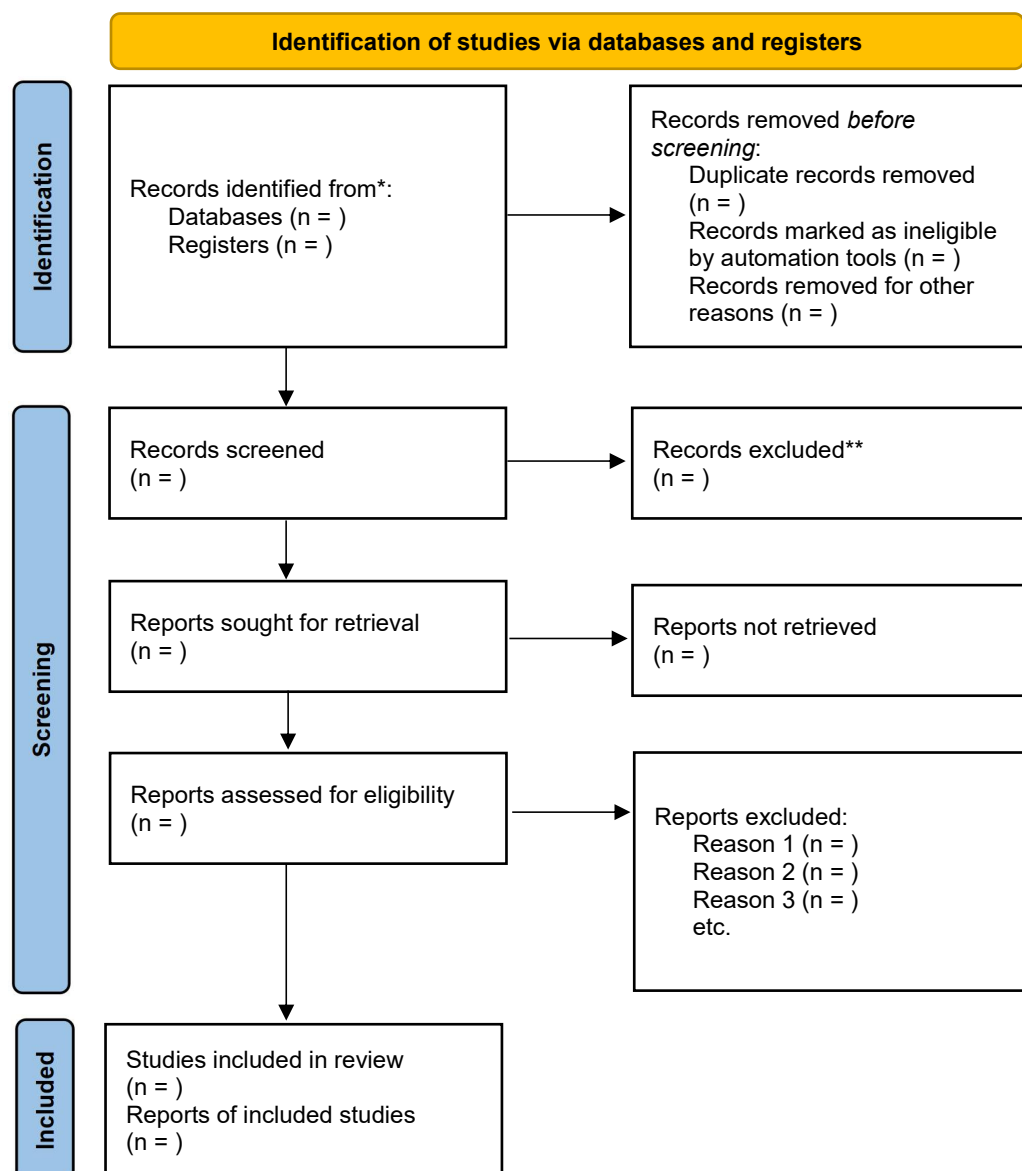

Supplement: Supplementary file 3 [file medi-102-e33247-s003.pdf]
